# Predictors of physical frailty improvement in older patients enrolled in a multidisciplinary cardiac rehabilitation program

**DOI:** 10.1007/s00380-023-02254-9

**Published:** 2023-03-30

**Authors:** Samuele Baldasseroni, Maria Vittoria Silverii, Andrea Herbst, Francesco Orso, Mauro Di Bari, Alessandra Pratesi, Costanza Burgisser, Andrea Ungar, Niccolò Marchionni, Francesco Fattirolli

**Affiliations:** Department of Experimental and Clinical Medicine, University of Florence and Cardiac Rehabilitation Unit, Azienda Ospedaliero-Universitaria Careggi, Largo Brambilla 3, 50134 Florence, Italy

**Keywords:** Elderly, Cardiac rehabilitation, Acute coronary syndrome, Short physical performance battery

## Abstract

Cardiac rehabilitation (CR) improves clinical and functional recovery in older patients after acute cardiac syndromes, whose outcome is influenced by cardiac disease severity, but also by comorbidity and frailty. The aim of the study was to analyze the predictors of physical frailty improvement during the CR program. Data were collected in all patients aged > 75 years consecutively admitted from 1 January to December 2017 to our CR, consisting of 5-day-per-week of 30-min session of biking or calisthenics on alternate days for 4 weeks. Physical frailty was measured with short physical performance battery (SPPB) at the entry and the end of CR. Outcome was represented by an increase of at least 1 point in the SPPB score from baseline to the end of the CR program. In our study population of 100 patients, mean age 81 years, we demonstrated that a strong predictor of improvement in SPPB score was the poorer performance in the test at baseline; for Δ-1 point of score, we registered an OR 2.50 (95% CI = 1.64–3.85; *p* = 0.001) of probability to improve the physical performance at the end of CR. Interestingly those patients with worse performance at SPPB balance and chair standing task showed greater probability of ameliorating their physical frailty profile at the end of CR. Our data strongly suggest that CR program after acute cardiac syndrome produces a significant physical frailty improvement in those patients with worse frailty phenotype with an impairment in chair standing or balance at entry.

## Introduction

The positive effect of cardiac rehabilitation (CR) has been clearly established both in young and older cardiovascular patients [1]. CR is a cornerstone of secondary prevention after cardiac disease [2], as it can improve short- and long-term survival [3] and, in the elderly, reduce the risk of morbidity and disability [4]. In the last decade, a dramatic change in the epidemiology of acute cardiac syndromes has been registered, which increasingly involves old and very old patients [5]. This implies a paradigm shift in the delivery of CR programs to this new type of patients.

Short- and long-term prognosis of older patients is undoubtedly influenced by severity of the cardiac disease per se, but also by the coexistence of complex comorbidity [6], global functional impairment, and different levels of cognitive and physical frailty [7]. Frailty, defined as an increased vulnerability to different acute stressors due to decreased physiological reserve [8], is widely recognized as a crucial clinical domain in older subjects, increasing the risk of disability, hospitalizations, morbidity and mortality [9]. In clinical practice, the operationalization of frailty is highly debated and follows two main approaches, one depicting frailty as a phenotype that can be captured by Fried’s criteria [10], and the other as a clinical state characterized by progressive accumulation of deficits, according to Rockwood’s model [11]. In substantial agreement with Fried’s model, and particularly in patients with cardiovascular diseases, frailty status is often assessed with physical performance measures, such as gait speed [12] or the short physical performance battery (SPPB) [13].

In spite of the compelling need for a comprehensive approach to older persons in CR, which should take into account the dimension of frailty, the efficacy of CR programs on frailty status in older patients has been poorly considered so far.

The aim of the present study was to register the effect of standardized CR after acute cardiac syndrome on physical frailty and which clinical and functional variables, routinely registered during our multidisciplinary rehabilitation program, showed an independent predictive value on SPPB score increase at the end of CR.

## Materials and methods

In an ancillary non-simultaneous cohort study design consisting of patients from the CR-AGE-Extra study [15], we considered all consecutive patients admitted from 1 January 2017 to 31 December 2017 to CR who received SPPB evaluation at the entry and the end of the program. Our study protocol was in agreement with the Declaration of Helsinki and was approved by our local ethics committee [15]; an informed consent was signed by all patients.

Our program has been described in detail elsewhere [15] and can be summarized as follows. The program consists of 5-day-per-week sessions of aerobic exercise for 4 weeks, at an intensity corresponding to 60–70% of peak VO_2_ consumption measured in a baseline, symptom-limited cardiopulmonary exercise test (CPET). This program duration reflects the length of CR usually provided by the Italian national health-care system. Each session consists of 30 min of either biking or calisthenics on alternate days, with an expert physiotherapist supervising activities through telemetric ECG and non-invasive arterial blood pressure monitoring.

Moderate-to-severe cognitive impairment (Mini-Mental State Examination score < 18) [16], disability in 2+ basic activities of daily living (BADL) [17], ejection fraction equal or less 35%, musculoskeletal diseases or other absolute contraindication to CPET, and diseases limiting life expectancy to < 6 months were taken as exclusion criteria [18].

According to a multidisciplinary approach, all patients were evaluated through a comprehensive geriatric assessment process, which included the definition of chronic comorbidity burden [19], independence in BADL, and psycho-emotional [20] and socio-economic profile by geriatricians, skilled nurses and physiotherapists. Loss of only one BADL and/or one or more instrumental ADL [21] was taken to indicate mild-to-moderate disability, but did not cause patient’s exclusion.

### Exercise capacity and muscle strength evaluation

As reported elsewhere [22], aerobic capacity was expressed as the peak VO_2_ consumption as resulting from breath-to-breath analysis (CPX Medical Graphics system) during a symptom-limited CPET on a cycle ergometer (Esaote Biomedica Formula). Changes in aerobic capacity at the end of the 4-week physical training were recorded. Muscle strength was measured at isokinetic dynamometer (BIODEX Medical System^®^) at three angular speeds (5 repeats at 90°/sec; 8 repeats at 120°/sec; 10 repeats at 180°/sec), evaluating the quadriceps and hamstring muscles strength, in flexion–extension of both inferior limbs. Submaximal exercise capacity was evaluated with the 6-min walking test according to the Guyatt’s protocol in a 30-m corridor [23] with telemetric ECG and O_2_ saturation monitoring, without previous familiarization test.

### Physical frailty profile

Participants underwent the SPPB according to standard procedures [24] at the beginning and at the end of CR. Total summary score, as well as results of each individual task [balance, gait speed, and repeated chair standing test), were recorded. The SPPB score was also categorized into an ordinal variable according to Guralnik’s grading of physical frailty [25]. A positive effect of CR was considered an increase in total SPPB score of at least 1 point from baseline to the end of CR [14].

### Statistical analysis

Data were analyzed using the SPSS 25.0 statistical package (SPSS, Inc., Chicago, IL). Admission characteristics were summarized with mean (SE) or frequency (%). Differences in clinical characteristics on admission between patients who did or did not achieve a substantial (at least 1 point) improvement in SPPB score were analyzed with Student’s *T* test or Chi square test as appropriate. Multivariable logistic regression models were built to identify the independent predictors of a substantial SPPB improvement and to calculate the corresponding odds ratios and 95% confidence interval (CI). The SPPB was entered as total score, categorized into four different levels, or as task subscores in separate logistic models.

A *p* value < 0.05 was considered as statistically significant.

## Results

### Baseline data

The study sample included 100 patients aged > 75 years (mean age 81 years; range 75–94), whose clinical characteristics are reported in Table 1. The average intervals from onset of the acute cardiac syndrome and from hospital discharge to enrollment were 26 ± 2 and 16 ± 2 days, respectively. In accordance with the exclusion criteria, 96% of patients were independent in 5 or more BADL and 74% had 6 or more IADL preserved, while the average cognitive and psycho-emotional profiles were good, as indicated by an MMSE score of 27.6 ± 0.3 and a 15-item GDS score of 3.8 ± 3.0. A Charlson Comorbidity Index of 6.0 ± 0.2 indicated an overall moderate burden of non-cardiovascular chronic comorbidity. Overall, the prescription rate of guidelines-recommended therapies after cardiovascular syndromes was satisfactory, particularly in the light of the advanced age of our study sample: in fact, 90% of the participants were treated with antiplatelets, 87% with beta-blockers, 81% with RAAS inhibitors, and 95% with statins.

**Table 1:** Baseline characteristics of the study participants

	*N* = 100
Age (years)	80.8 ± 0.5
Male gender	80 (80.0)
BMI (kg/m^2^)	27.2 ± 0.4
Hypertension	78 (78.0)
Diabetes	20 (20.0)
Dyslipidemia	56 (56.0)
Current smoking	14 (14.0)
COPD	13 (13.0)
Preserved BADL	5.6 ± 0.1
Preserved IADL	6.3 ± 0.2
Charlson Comorbidity Index	6.0 ± 0.2
MMSE score	27.7 ± 0.3
GDS score	3.8 ± 0.3
SPBB total score	9.4 ± 0.2
Cardiovascular syndromes	
NSTEMI	36 (%)
STEMI	31 (%)
Valvular surgery	16 (%)
CABG	17 (%)
Hemoglobin	12.0 ± 0.2
CKD-epi GFR	59.6 ± 2.0
LVEF (%)	50.5 ± 1.0
6-min walking test (mt)	393.8 ± 11.3
90° Torque peak (N × mt)	53.9 ± 2.5
Peak VO_2_ consumption (ml/kg/min)	13.9 ± 0.4

Data are mean ± SE or *n* (%)

*BMI* body mass index, *COPD* chronic obstructive pulmonary disease, *BADL/IADL* basic/instrumental activities of daily living, *MMSE* Mini-Mental State Examination, *GDS* geriatric depression scale, *RAAS* renin–angiotensin–aldosterone system, *NSTEMI-UA* non-ST segment elevation myocardial infarction/unstable angina, *STEMI* ST segment elevation myocardial infarction, *LVEF* left ventricular ejection fraction

### Exercise capacity and muscle strength

At the end of the CR program, a marked improvement in aerobic exercise capacity was observed, as shown by a statistically significant increase in mean VO_2_ peak at the end of the CR program (baseline:13.8 ± 0.4 ml/kg/min vs. end of CR: 14.7 ± 0.4 ml/kg/min; *p* < 0.001). Similar findings were obtained also for other functional measures: the total distance walked in 6 min increased from 392.6 ± 12.0 to 410.1 ± 12.1 m (*p* < 0.001), whereas muscle strength improved from 52.6 ± 2.7 to 63.1 ± 2.8 N × m (*p* < 0.001).

### Physical frailty phenotype

**Fig. 1:**
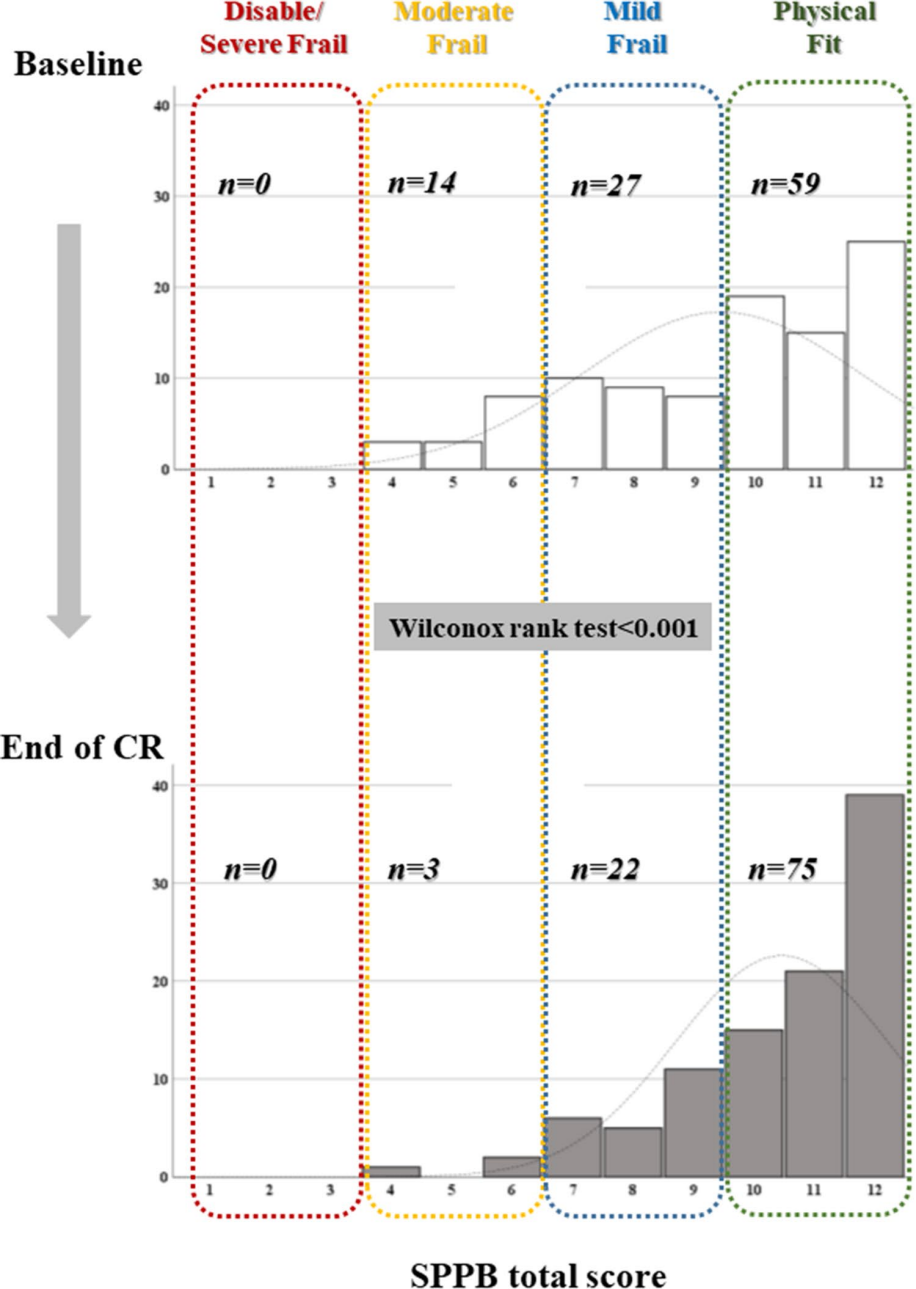
Improvement of physical frailty phenotype from the entry to the end of the CR program

**Fig. 2:**
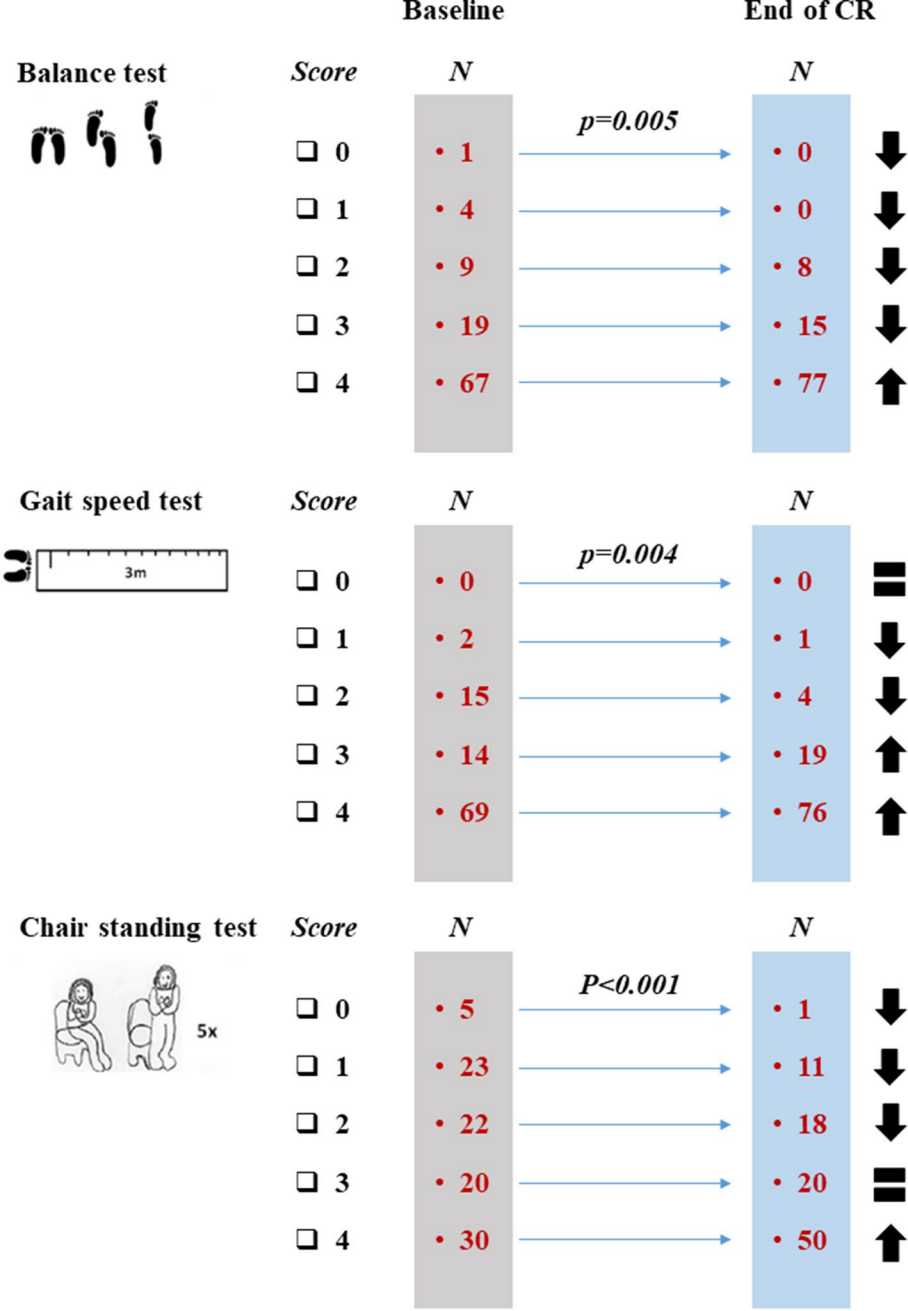
Improvement in the three subscale task scores of SPPB from the entry to the end of the CR program

In parallel, after the CR program, we observed an increase in SPPB total score, from 9.4 ± 0.2 to 10.5 ± 0.3 (*p* < 0.001); 48 participants (48%) obtained at least 1 point increase in SPPB total score at the end of CR, 47 participants (47%) remained unchanged, and only 5 (5%) declined. As shown in Figs. 1 and 2, the frailty phenotype shift toward improving at the end of CR was balanced across the SPPB task subscores.

### Determinants of physical frailty improvement

#### Univariate analysis

At baseline, the distribution of SPPB score in the study population was as follows: a total score equal to or less than 10 points in 41 patients (41%); 10 points in 19 patients (19%), more than 10 points in 40 patients (40%). The number of patients in whom the SPPB total score remained unchanged at the end of CR was 61 (61%). Among those changed their performance at SPPB, 32 (78%) patients among those scored < 10 points at baseline reached 1 point of increase in SPPB total score. Among forty patients with baseline score > 10 points, 7 (17.5%) reached 1 point of increase.

Differences in clinical and functional parameters between participants whose SPPB total score remained unchanged or worsened at the end of the CR program (Group A) and those in whom it increased by at least 1 point (Group B) are shown in Table 2. Notably, at baseline, Group B participants had worse physical frailty status, cardiorespiratory performance and muscle strength.

**Table 2:** Baseline variables significantly associated with 1+ point increase in SPPB total score at the end of the CR program

Variables	Group A *N* = 52	Group B *N* = 48	*p* value
SPPB total score	10.7 ± 0.2	8.1 ± 0.3	< 0.001
Gait speed test (score)	3.8 ± 0.1	3.2 ± 0.1	0.002
Chair stand test (score)	3.2 ± 0.1	1.7 ± 0.2	< 0.001
Balance test (score)	3.8 ± 0.1	3.1 ± 0.2	< 0.001
SPPB categorized			
Disabled/severely frail (*n*)	0 (0)	0 (0)	< 0.001
Moderately frail (*n*)	1 (1.9)	13 (27.1)	
Mildly frail (*n*)	8 (15.4)	19 (39.6)	
Not frail (*n*)	43 (82.7)	16 (33.3)	
No. of preserved IADL	6.7 ± 0.2	5.9 ± 0.3	0.030
6-min walking test (mt)	421.1 ± 15.2	362.3 ± 15.6	0.009
90° torque peak (N × mt)	61.0 ± 3.6	46.1 ± 3.1	0.003
Peak VO_2_ consumption (ml/kg/min)	14.6 ± 0.5	13.2 ± 0.4	0.050
Female gender	8 (14)	12 (25)	0.230
BMI (kg/h^2^)	26.9 ± 4.6	25.8 ± 4.4	0.124
Diabetes	10 (19.3)	16 (33.3)	0.108
Dyslipidemia	30 (52.7)	26 (54.2)	0.723
COPD	6 (11.5)	7 (14.6)	0.651
No. of preserved BADL	5.6 ± 0.5	5.6 ± 0.7	0.416
MMSE score	27.5 ± 2.3	27.8 ± 2.8	0.274
GDS score	3.6 ± 3.3	3.9 ± 3.1	0.345
LVEF	50.5 ± 10.2	50.6 ± 10.3	0.448
Charlson Comorbidity Index score	6.0 ± 2.1	6.0 ± 2.0	0.441
Hemoglobin (gr/dl)	12.1 ± 1.7	11.1 ± 1.6	0.253

*Group A* unchanged or worsened SPPB total score, *Group B* at least 1-point increase in SPPB total score. Data are mean ± SE or *n* (%)

Abbreviations as in Table 1

#### Multivariate analysis

The results of multivariable models to identify the independent predictors of a substantial SPPB improvement are reported in Table 3. The strongest predictor of improvement was a more severe frailty status at baseline: this finding was not influenced by modeling of the SPPB score in the logistic regression analysis, as it was similar when it was entered as a continuous variable or categorized as an ordinal variable. In the model where the three subscores were entered separately, baseline balance and chair standing tests seemed to be the best independent predictors of a positive shift in frailty status.

**Table 3:** Baseline independent predictors of improvement in physical frailty status, defined as an at least 1-point increase in SPPB total score

Variables	OR	95% CI	*p* value
Model 1, (*R*^2^ Nabelkerke = 0.49)			
IADL preserved	0.98	0.68–1.42	0.925
6-min walking test (1 mt)	1.01	1.00–1.02	0.045
90° torque peak (1 N × mt)	0.98	0.95–1.00	0.100
Peak VO_2_ consumption (1 ml/kg/min)	0.99	0.80–1.23	0.911
SPPB total score (Δ-1 point)	2.50	1.64–3.85	0.001
Model 2, (*R*^2^ Nabelkerke = 0.39)			
IADL preserved	0.76	0.67–1.34	0.759
6-min walking test	1.01	0.99–1.01	0.198
90° torque peak	0.98	0.95–1.00	0.099
Peak VO_2_ consumption	0.96	0.79–1.17	0.690
SPPB category (moderately frail vs. others)	7.96	2.70–25.00	0.001
Model 3, (*R*^2^ Nabelkerke = 0.51)			
IADL preserved	1.01	0.69–1.49	0.947
6-min walking test	1.01	1.00–1.02	0.060
90° torque peak	0.98	0.95–1.01	0.098
Peak VO_2_ consumption	1.00	0.81–1.24	0.990
SPPB balance test score (Δ-1 point)	3.16	1.10–9.09	0.032
SPPB gait speed test score (Δ-1 point)	1.37	0.60–3.16	0.449
SPPB chair standing test score (Δ-1 point)	3.26	1.72–5.88	0.001

MODEL 1: SPPB introduced as an interval variable

MODEL 2: SPPB introduced as a categorized variable

MODEL 3, SPPB introduced as task subscores

Abbreviations as in Table 1

## Discussion

Our findings, obtained with a structured multidisciplinary CR started soon after an acute cardiac event in a very old (mean age 81 years) population, can be summarized as follows. First, a CR program based on 5-day-per-week sessions for 4 weeks induced a significant improvement in frailty status, defined as 1 point of increase in SPPB total score [14], in almost 50% of our participants after acute cardiac syndrome; 47% of them maintained stable physical performance at SPPB evaluation and only 5% lost 1 point at the end of CR. Second, the improvement in SPPB evaluation was consistent across all the three SPPB tasks. Third, functional benefit from CR was obtained in participants whose baseline global SPPB performance, expressed either as a continuous or a categorical score, was poorer; of the three SPPB tasks, only the result on balance and chair standing tests was independently associated with improvement at the end of CR.

In a recent retrospective analysis of 243 patients with cardiac disease, Lutz and Coll. [26] demonstrated that a CR program was able to provide improvements in multiple aspects of physical functioning, and gains achieved by frail adults were at least comparable to, or even greater than, those classified as robust. Among participants that presented with different levels of frailty on admission to CR, we registered higher functional benefit in those with an initially more compromised frailty status. This finding is consistent with a large study (*n* = 2322) by Kehler et al. [27], which demonstrated that completion of a CR program was associated with lower frailty levels, as assessed from Rockwood’s accumulation of deficit approach: after adjustment for age, sex, and number of exercise sessions attended, frailty status improved in all frail groups, in particular in the frailest subjects. Our findings compare well with those reached in older subjects examined by Rinaldi et al. [14], although our sample was almost 15 years older than Rinaldi’s. Thus, taken together, these data seem to suggest that the probability of obtaining a significant functional benefit depends more on the frailty level than to age per se. Furthermore, all these data provide support for referring all eligible cardiac patients to CR regardless of frailty status, and possibly favoring those who are frailer when CR resources are limited.

In addition, our findings should stimulate researchers to develop CR programs tailored to frail older patients, whereas such programs are commonly more oriented toward younger patients. Moreover, it might be suggested to individualize CR programs based on the results of the SPPB at the entry, considering both the summary score and the three different SPPB tasks. In this perspective, recent data from the REHAB-HF trial [28] showed how in older adults recently hospitalized for acute decompensated heart failure, a multiple physical function-adapted rehabilitation program initiated during, or early after, hospitalization for heart failure and continued after discharge for 36 outpatient sessions reached substantial improvements in global physical function compared to usual care.

This result was obtained with an early, transitional, tailored, progressive physical rehabilitation program that had been developed for frail, older patients with acute decompensated heart failure. The REHAB-HF program focused on four physical function domains (strength, balance, mobility, and endurance); the progression of exercise intensity and the types of exercises at each session were individualized on the basis of the patient’s performance level within each domain and a key goal was to increase each patient’s exercise endurance (duration of walking). At the end of the program, the intervention group obtained a statistically significant improvement in SPPB score with respect to the control group.

Even in our cohort study, frailty level improved or at least stabilized at the end of the CR program, a clinically relevant finding in a geriatric perspective. Kim et al. [29] recently demonstrated that functional status and frailty take different trajectories after a stressful cardiac event (i.e., surgery or transcatheter aortic valve replacement): patients with higher functional status before the procedure had a greater probability of recovering their preoperative function, whereas those with moderate or severe preoperative frailty status had poor or very poor postoperative trajectories and often remained persistently impaired. Moreover, functional trajectories were significantly influenced by procedure type, preoperative frailty, and postoperative complications. Thus, the authors strongly underlined the crucial role of rehabilitation program soon after the intervention in the hope of modifying positively the poor trajectories of frail patients. Differently, we found in univariate analysis that those patients with lower number of IADLs preserved seem to have more probability of gaining a physical functional benefit from CR, though this association was lost in multivariable analysis in part explainable by a small sample size.

In a geriatric continuum care perspective, data from Molino Lova et al. [30] suggested that a long-term exercise program including exercises for strength, flexibility, balance and coordination, conducted under a physiotherapist’s supervision and associated with planned reinforcing follow-up visits, may successfully counteract, or at least slow down, the decline in physical functioning in older patients remaining frail after conclusion of a CR program.

Another interesting finding of present study is that the results of balance and chair standing tests at the entry are more powerful predictor of frailty improvement at the end of CR in respect of Gait speed test one. This novel finding leads us to raise two clinical considerations. First, as already underlined by Guralnik [31], the SPPB test appears as a more accurate measure of physical frailty than gait speed. In fact, the battery explores more aspects of lower extremity performance, involved in postural response and balance recovery after a rapid body displacement, which depends more on central and peripheral nervous system activity than purely on muscle strength. Thus, the SPPB explores more extensively the homeostatic reserve of different biological systems after a stressful disturbance, in good agreement with the definition of frailty [8]. Secondly, as suggested by Verbrugge et al. [32], it is possible that the chair and balance tests give information on the pathway from cardiac disease to frailty and subsequent disability. We would hypothesize that, in older patients, acute cardiac event could impact more significantly these two domains than on gait speed, confirming Verbrugge’s assumption [32] that specific diseases may affect differently lower extremity functioning after an acute event, such as hospitalization for acute cardiac disease.

Finally, it is clearly established that a low level of physical functional capacity before cardiac surgery is able to influence negatively postoperative outcomes, such as length of hospital stay, major morbidity, and mortality [33], as well as patient-centered outcomes, such as quality of life [34].

### Study limitations

The study presents the limitation of the non-randomized control study. In addition, we cannot exclude a partial contribution of ceiling effect on SPPB improvement in those participants with lower scores than those with higher scores at CR entry. The exclusion of individuals with severe cognitive decline or severe physical frailty/disability might limit the generalizability of our findings to the broader spectrum of older adults routinely hospitalized for acute cardiac syndromes, although these patients often presented contraindications to referral for CR. Our CR program was relatively brief, as 4 weeks may be regarded as insufficient to maximize the possible improvement in frailty level. However, this duration reflects the routine length of rehabilitation provided by the Italian national health-care system and, therefore, our study provides information that is relevant in the perspective of real-world rehabilitation practice in Italy. Another statistical limitation is related to the clinical value of 1 point increase in SPPB in the presence of different levels of frailty at baseline, but unfortunately our sample size did not allow subgroup analysis.

## Conclusion

The crucial role of CR is clearly established in secondary prevention after an acute cardiac syndrome, and different modalities of CR programs may provide positive results. However, all these programs are usually targeted to recover from the consequences of cardiac disease per se and to guarantee the best cardiorespiratory exercise capacity, according to a purely cardiologic perspective that ignores age-related frailty. Together with other recent clinical observations, our data strongly suggest that CR has positive and significant benefit on global functional capacity, particularly in those with a compromised frailty phenotype. Should future large randomized trials confirm these evidence from our observational study in the setting of acute cardiac syndrome, these would undoubtedly encourage planning different types of CR, more tailored to geriatric patients and domains as recently shown in REHAB-HF study for patients after acute decompensated heart failure. Given the prevalence of oldest old subjects with acute cardiac disease, who are increasingly found to be candidates for complex cardiological interventions, such randomized controlled studies are urgently needed.

## Data Availability

We confirm the availability of clinical data of patients enrolled in our database as well as their availability for review by an external auditor.
